# Trends in bariatric surgeries in the Brazilian Federative Units, 2009-2019: a descriptive study

**DOI:** 10.1590/0100-6991e-20223335-en

**Published:** 2022-10-26

**Authors:** VINÍCIUS DA SILVA OLIVEIRA, VINÍCIUS BARROS CHAVES, ARTHUR ADIB NERY ABOUD, ANELIZE MARIA BUNHOLLI, RAFAEL MENDONÇA MACEDO, RENATA MACHADO PINTO

**Affiliations:** 1 - Universidade Federal de Goiás - Goiânia - GO - Brasil; 2 - Universidade Federal de Goiás, Departamento de Pediatria - Goiânia - GO - Brasil

**Keywords:** Health Services Accessibility, Bariatric Surgery, Time Series Studies, Epidemiology, Obesity Management, Acesso aos Serviços de Saúde, Cirurgia Bariátrica, Estudos de Séries Temporais, Epidemiologia, Manejo da Obesidade

## Abstract

**Objective:**

our objective is to describe the epidemiological distribution of hospitalizations and postoperative deaths, as well as the trends of bariatric surgeries performed by SUS in all Brazilian federative units (FUs) from an analysis of the period from 2009 to 2019.

**Methods:**

This is an observational, descriptive ecological time-series study with quantitative and descriptive analysis, based on secondary data. The period analyzed was from 2009 to 2019. We collected, from DATASUS, data from obese men and women who were hospitalized after undergoing bariatric surgery. Prais-Winsten regression was performed to identify the trends.

**Results:**

In the period, 83,829 bariatric surgeries were performed, of which 161 resulted in death, representing 0.19% of the procedures. We found an increasing trend in the number of surgeries for Brazil (β=0.04; p<0.001), but 11 FUs showed a stationary trend and three, decreasing ones (six UFs did not have enough data to enter the analysis). In the North and Northeast regions, stationary trends prevailed, while in the Center-West, the decreasing trends, and in the South and Southeast, the increasing ones.

**Conclusions:**

we found an evident disparity between regions, suggesting deficiencies in access to health. By demonstrating which FUs and demographic characteristics have the lowest rates of surgeries, our study is able to direct public policies towards a more egalitarian Brazilian public health.

## RESUMO

**Objetivo:**

nosso objetivo é descrever a distribuição epidemiológica das internações e óbitos pós-operatórios, bem como as tendências das cirurgias bariátricas realizadas pelo SUS em todas as unidades federativas (UFs) brasileiras a partir de uma análise do período de 2009 a 2019.

**Métodos:**

trata-se de um estudo observacional descritivo ecológico de série temporal com análise quantitativa e descritiva, com base em dados secundários. O período analisado foi de 2009 a 2019. Coletamos, no DATASUS, dados de homens e mulheres que possuem obesidade e foram internados após terem sido submetidos à cirurgia bariátrica. A regressão de Prais-Winsten foi utilizada para identificar as tendências.

**Resultados:**

no período, 83.829 cirurgias bariátricas foram realizadas, tendo 161 evoluído para óbitos, o que representa 0,19% dos procedimentos. Encontramos uma tendência crescente no número de cirurgias para o Brasil (β=0,04; p<0,001), mas 11 UFs apresentaram tendência estacionária e três, decrescente (seis UFs não tinham dados suficientes para entrar na análise). Nas regiões Norte e Nordeste prevaleceram tendências estacionárias, enquanto no Centro-Oeste, as decrescentes, e no Sul e Sudeste, as crescentes.

**Conclusões:**

encontramos uma evidente disparidade entre as regiões, sugerindo deficiências de acesso à saúde. Ao demonstrar quais UFs e características demográficas apresentam menores índices de cirurgias, nosso estudo é capaz de direcionar políticas públicas para uma saúde pública brasileira mais igualitária.

## INTRODUCTION

Obesity is considered a pandemic disease that affects more than 650 million people worldwide^1^. In severe cases, bariatric surgery is the most effective therapeutic option, leading to a better quality of life and increased life expectancy^2^ ^,^ ^3^. In Brazil, overweight and obesity reached, respectively, 55.4% and 20.3% of adults in the states’ capitals in 2019^4^. Furthermore, the increase in overweight and obesity in the country is evident in all age groups, sexes, and social classes, especially in the poorest population^5^.

Non-surgical therapeutic options for obesity include lifestyle changes, regular physical activity, dietary reeducation, and use of drugs with malabsorptive, satiety, and/or anorectic effects^6^. However, surgical treatment is a good adjunct to the management of the disease and comorbidities. A systematic review with meta-analysis with 174,772 participants found that metabolic/bariatric surgery was associated with a 49.2% reduction in risk of death compared with the usual obesity management^7^. Surgery has also been effective in controlling type 2 diabetes, hypertension, and weight loss^8^ ^-^ ^10^.

The most performed surgeries around the world are sleeve gastrectomy (Sleeve), Roux-en-Y Gastroplasty, Adjustable Gastric Band, and Duodenal Switch^2^. Surgery involves investigating the patient’s clinical history and progression of obesity. Weight loss peaks at two years of follow-up and remains relatively stable over the next few years^11^.

In Brazil, obesity has become the focus of public policies in the last 15 years, which has led the Ministry of Health to become the main proponent of strategies to combat this chronic disease^12^. Bariatric surgery is indicated for people with at least 16 years of age and BMI greater than 50, with BMI greater than 40 and unsuccessful clinical treatment, and over 35 years old with comorbidities^13^. In 2017, the Brazilian Public Unified Health System (SUS) introduced the laparoscopic bariatric procedure as one of the possible options for performing the surgery^14^.

Due to the high prevalence of obesity and the absence in the literature of studies on the trend of bariatric surgeries in Brazil, the present study is necessary. Thus, our objective is to describe the epidemiological distribution of hospitalizations and postoperative deaths, as well as the trends of bariatric surgeries performed by the SUS in all Brazilian Federative Units (FUs), in the period from 2009 to 2019. Prior to the development of the study, we hypothesized that we would find heterogeneity among the FUs’ trends, with more growing trends in the states of the South and Southeast regions, in addition to a stationary trend for the country.

## METHODS

### Study project and participants

This is an observational, descriptive, ecological, time series study, with quantitative and descriptive analysis, based on secondary data.

Data were collected in March 2021, from the Hospital Information System (SIH-SUS) and from the Population Projection of the Federation Units by sex and age groups (2000 2030), both made available by DATASUS, which is the informatics department of the Brazilian Unified Health System. It is a national agency of the Ministry of Health, which is responsible for collecting, processing, and disseminating health information.

The study analyzed data and characteristics of men and women of all ages undergoing bariatric surgery with resources from SUS in all 27 Brazilian FUs. The country has 26 states and one Federal District, which are divided into five geographic regions: North, Northeast, Midwest, Southeast, and South.

To identify the cases, we tabulated the hospitalizations in the SIH-SUS, which was accessed by DATASUS Tabwin. The tabulation comprised patients with obesity (ICD-10 E66) who underwent the procedure authorized by SUS to treat this disease, under the codes below:

04.07.01.012-2: Gastrectomy with or without duodenal deviation;

04.07.01.036-0: Sleeve gastrectomy;

04.07.01.017-3: Gastroplasty with intestinal bypass;

04.07.01.018-1: Vertical gastroplasty with band;

04.07.01.038-6: Laparoscopic bariatric surgery.

To calculate the rates, the population estimate was taken from the Population Projection of the Federation Units by sex and age groups (2000 2030).

Data referred to the period from 2009 to 2019.

### Variables

Initially, a descriptive analysis of the sample was performed using absolute and relative frequencies. The following qualitative variables were tabulated: sex (Male/Female); age group; race/color (White/Black/Brown/Yellow/Indigenous); procedure performed (Gastrectomy with or without duodenal bypass/Sleeve Gastrectomy/Gastric bypass/Vertical gastroplasty with band/Laparoscopic bariatric surgery); hospitalization regimen (Public/Private); and Geographic Region (North/Northeast/Midwest/South/Southeast).

### Statistical methods

For each variable, we describe the absolute and relative frequencies of both surgeries and deaths, in addition to the total value (in Brazilian Reais - R$) and average value (also in Reais) spent on the procedures.

With hospitalization data and population data, we calculated the Hospitalization Rates (HR) with the following formula:

We calculated the base 10 logarithms of these rates to perform the Prais-Winsten regression, identifying trends in hospitalizations through the slope coefficient of the line formed during the regression, which is increasing for positive coefficients and decreasing for negative coefficients, when significant. We considered p<0.05 as statistically significant.

The FUs with less than five years of data did not participate in the time series analysis to avoid bias, but we used their data to present the general panorama of the country in the descriptive analysis.

Data were tabulated in the Excel software and analyzes were performed in the Stata version 14.0 (StataCorp. 2013. Stata Statistical Software: Release 13. College Station, TX: StataCorp LP).

### Ethical aspects

As this study used secondary data without identifying the participants, it was not necessary to submit it to the Ethics in Research Committee, as determined by Resolution of the National Health Council (CNS) No. 466, of December 12, 2012, and Resolution CNS No. 510, dated April 7, 2016^15^ ^,^ ^16^.

## RESULTS

### Participants and descriptive data

Between 2009 and 2019, 83,829 bariatric surgeries were performed for the treatment of obesity funded by SUS in Brazil. Table 1 describes the characteristics of these hospitalizations, showing that most patients were female (85.0%), white (65.2%), and aged between 35 and 44 years (33.1%). The main procedure was the Roux-en-Y Gastric Bypass (93.7%). In SUS, hospitalizations and surgical procedures can be performed in public hospitals, or they can be subsidized by the System in private institutions. In this study, the hospitalization regimen, when identified, was mainly in private hospitals (35.6%). The total amount spent in the period was R$ 514,110,337.00, with distribution proportional to the population characteristics.

**Table 1:** Demographic and socioeconomic characteristics of hospitalizations for bariatric surgeries in Brazil between 2009 and 2019.

Variable	Hospitalizations n (%)	Amount R$ (%)	Relative value R$	Deaths n (%)
Sex				
Male	12,549 (15.0)	78,012,889 (15.2)	6,216.66	56 (34.8)
Female	71,280 (85.0)	436,097,448 (84.8)	6,118.09	105 (65.2)
Race/color				
White	54,661 (65.2)	336,822,329 (65.5)	6,162.02	89 (55.3)
Black	3,427 (4.1)	20,894,032 (4.1)	6,096.89	5 (3.1)
Brown	15,205 (18.1)	93,709,060 (18.2)	6,163.04	45 (28.0)
Yellow	533 (0.6)	3,252,211 (0.6)	6,101.70	0 (0.0)
Indigenous	13 (<0.1)	79,207 (<0.1)	6,092.84	1 (0.6)
No information	9,990 (11.9)	59,353,498 (11.5)	5,941.29	21 (13.0)
Age group				
<15 years	10 (<0.1)	55,037 (<0.1)	5,503.70	0
15 - 24 years	6,061 (7.2)	37,117,451 (7.2)	6,123.98	4 (2.5)
25 - 34 years	24,519 (29.2)	149,365,793 (29.1)	6,091.84	24 (14.9)
Variable	Hospitalizations n (%)	Amount R$ (%)	Relative value R$	Deaths n (%)
35 - 44 years	27,784 (33.1)	170,076,433 (33.1)	6,121.38	42 (26.1)
45 - 54 years	17,745 (21.2)	109,231,042 (21.2)	6,155.60	40 (24.8)
55 - 64 years	7,076 (8.4)	44,121,349 (8.6)	6,235.35	41 (25.4)
≥65 years	634 (0.8)	4,143,230 (0.8)	6,535.06	10 (6.3)
Procedure				
Gastrectomy with or without duodenal bypass	405 (0.5)	2,473,712 (0.5)	6,107.93	10 (6.2)
Gastroplasty with intestinal bypass	78,583 (93.7)	483,872,510 (94.1)	6,157.47	144 (89.4)
Vertical gastroplasty with band	1,282 (1.5)	6,034,545 (1.2)	4,707.13	4 (2.5)
Sleeve gastrectomy	2009 (2.4)	12,038,247 (2.3)	5,992.15	2 (1,2)
Laparoscopic bariatric surgery	1,550 (1.9)	9,691,323 (1.9)	6,252.46	1 (0.6)
Regimen				
Public	9,737 (11.6)	55,543,559 (10.8)	5,704.38	26 (16.1)
Private	29,817 (35.6)	175,623,085 (34.2)	5,890.03	65 (40.4)
Unknown	44,275 (52.8)	282,943,693 (55.0)	6,390.59	70 (43.5)
Region				
North	697 (0.8)	4,115,723 (0.8)	5,904.91	3 (1.9)
Northeast	5662 (6.8)	33,794,889 (6.6)	5,968.72	5 (3.1)
Southeast	27342 (32.6)	163,195,519 (31.7)	5,968.68	60 (37.3)
South	48782 (58.2)	304,974,876 (59.3)	6,251.79	84 (52.2)
Midwest	1346 (1.6)	8,029,329 (1.6)	5,965.33	9 (5.6)
TOTAL	83,829 (100.0)	514,110,337 (100.0)	6,132.85	161 (100.0)

The number of deaths was 161, which represents only 0.19% of the procedures. The majority occurred under the gastric bypass procedure code (89.4%), in female patients (65.2%), and with white skin (55.3%). When the hospitalization regimen was known, there was a higher proportion of deaths in the private health system (40.4%). 

### Main results

The average hospitalization rates per 100,000 inhabitants ranged from zero (in the states of Rondônia, Amazonas, Roraima, Amapá, and Piauí) to 43.29 (in the state of Paraná). The average rate for Brazil was 4.86. The time series analysis revealed an increasing trend for the country (β=0.04, p<0.001), although 11 states showed stationary trends. In addition, seven states showed increasing trends and three showed decreasing trends (Table 2).

**Table 2:** Time series (2009-2019) and trends in SUS-funded bariatric surgeries in Brazilian Federative Units.

Federative unit	Coefficient	Confidence Interval 95%	p-value	Trend
North				
Acre	0.39	[-0.19, 0.27]	0.70	Stationary
Amapá	-	-	-	-
Amazon	-	-	-	-
Pará	-0.03	[-0.10, 0.04]	0.41	Stationary
Rondônia	-	-	-	-
Roraima	-	-	-	-
Tocantins	0.26	[-0.01, 0.07]	0.19	Stationary
Northeast				
Alagoas	0.02	[-0.01, 0.06]	0.19	Stationary
Bahia	-0.09	[-0.18, -0.01]	0.03*	Descending
Ceará	0.01	[0.00, 0.02]	0.10	Stationary
Maranhão	0.15	[0.11, 0.19]	<0.001*	Increasing
Paraíba	0.12	[0.05, 0.18]	0.01*	Increasing
Pernambuco	0.00	[-0.03, 0.02]	0.71	Stationary
Piauí	-	-	-	-
Rio Grande do Norte	0.22	[-0.03, 0.07]	0.32	Stationary
Sergipe	0.02	[-0.01, 0.04]	0.23	Stationary
Southeast				
Espírito Santo	0.06	[0.04, 0.08]	<0.001*	Increasing
Minas Gerais	0.11	[0.08, 0.15]	<0.001*	Increasing
Rio de Janeiro	0.04	[0.00, 0.08]	0.04*	Increasing
São Paulo	0.01	[-0.01, 0.03]	0.30	Stationary
South				
Paraná	0.06	[0.05, 0.08]	<0.001*	Increasing
Rio Grande do Sul	0.03	[0.02, 0.04]	<0.001*	Increasing
Santa Catarina	0.01	[-0.01, 0.03]	0.19	Stationary
Midwest				
Federal District	-0.11	[-0.20, -0.01]	0.03*	Descending
Goiás	-	-	-	-
Mato Grosso	-0.30	[-0.46, -0.15]	0.01*	Descending
Mato Grosso do Sul	-0.08	[-0.20, 0.04]	0.17	Stationary
Brazil	0.04	[0.04, 0.05]	<0.001*	Increasing

*Statistically significant

Six FUs (Amapá, Amazonas, Goiás, Piauí, Rondônia, and Roraima) could not participate in the analysis because they did not have data for at least five years. Of these six FUs, Goiás could not participate in the time series analysis because it only presented hospitalizations in the last two years (2018 and 2019), an insufficient period for the analysis. The other five states did not have any reported hospitalizations during the entire study period and, therefore, were excluded from the time series analysis.

Most states in the North and Northeast showed stationary trends. In the North region, all were stationary, with an average rate of 0.75 hospitalizations/ 100,000 inhabitants. In the Northeast region, the states of Maranhão (β=0.15, p<0.001) and Paraíba (β=0.12, p=0.01) showed growing trends, while Bahia (β=-0.09, p=0.03) showed a decreasing one. The average rate for the region was 1.24 hospitalizations/100,000 inhabitants.

The Southeast and South regions had average rates of 3.68 and 19.08 hospitalizations/ 100,000 inhabitants, respectively. Most of the states of both regions presented with increasing trends and no states with decreasing ones. Stationary trends occurred only in São Paulo and Santa Catarina. In the southern region, the state of Paraná showed an increasing trend (β=0.06, p=<0.001), having performed 41,804 bariatric surgeries in the period, accounting alone for 49.9% of the surgeries in the country.

Finally, in the Midwest region, the Federal District (β=-0.11, p=0.03) and Mato Grosso (β=-0.30, p=0.01) showed decreasing trends, while Mato Grosso do Sul displayed a stationary trend.

## DISCUSSION

Regarding hospitalizations, the group with the highest prevalence was female, white, and aged between 35 and 44 years. The state of Paraná lead, with 49.9% of surgeries. The most performed procedure was gastroplasty with intestinal bypass. It is noteworthy that the hospital regimen was unknown in more than half of the cases. Deaths were statistically associated with male sex, brown skin, indigenous race, and older age. We found an increasing trend in the number of surgeries for Brazil, but 11 FUs showed a stationary trend, and three, a decreasing one (six UFs did not have enough data to be assessed). In the North and Northeast regions, stationary trends prevailed, while in the Midwest, the decreasing trends, and in the South and Southeast, the increasing ones.

Obese women have greater body dissatisfaction when compared with men^17^, which may explain, at least in part, the significant difference in surgeries performed on women (85% versus 15%), despite the similar prevalence of the disease between sexes. The highest proportion of bariatric surgeries in the female population is not exclusive to Brazil: in the United Kingdom, for example, between 2012 and 2014, 75% of these surgeries were performed on women^18^. In addition, historically there is a lower demand for health services by men, which explains, at least in part, the higher life expectancy of women^19^. Thus, as bariatric surgery is a therapeutic intervention for a health problem, we hypothesize that women seek more treatment.

When analyzing the relationship between race, we found that deaths and hospitalizations due to bariatric surgery in white people (55.3% and 65.2%, respectively) are much higher than in blacks (3.1% and 4.1%) and brown (28.0% and 18.1%). These data do not correspond to the race proportions of the overweight and obese population4 and are probably due to unequal access and quality of health services among different ethnicities, since brown and black skin are factors associated with difficult access to health care in Brazil^20^.

Regarding age groups, people between 35 and 44 years old were the ones who underwent most bariatric procedures in Brazil during the study period (33.1%), while the age groups associated with the highest mortality were those between 55 and 64 and 65 years or older, possibly due to more complications caused by age and comorbidities. However, even at this age, bariatric surgery can still be performed, as long as there is greater medical follow-up with criteria segregated by different specialties, as these patients are more likely to have chronic diseases, such as hypertension and diabetes^21^ ^,^ ^22^.

Bariatric surgery also contributes to the remission of these chronic diseases, and it is necessary to evaluate the risks and benefits of this procedure in such patients. According to the literature, the surgical approach is more efficient in terms of weight loss and maintenance, reduced mortality, and control of diabetes and hypertension^7^ ^-^ ^10^.

Regarding the difference in expenses for bariatric surgery in public and private hospitals, the average values of the procedures are similar, but there are fewer hospitalizations in public institutions. This raises the issue that investment should be directed to the public hospitals, validating the organic laws of the Unified Health Service, in which the private network can be targeted by SUS investment but will act in a complementary way and not as the main setting for health treatment of the population^23^. Underreporting is also noteworthy, as the hospital regimen was unknown in more than half of the cases. Therefore, the real situation becomes unknown, which makes it difficult for the public manager to take effective actions and generates unnecessary expenses that could be better used in health care. Thus, the complementary role of private health in relation to SUS is impaired, which implies the under-achievement of its principles and guidelines, as well as alerting to an in-progress counter-reform of the system.

Moreover, as for the different techniques applied, the most performed procedure was gastroplasty with intestinal bypass (gastric bypass). Even so, laparoscopic surgery, associated with lower complication rates, less postoperative pain, and faster recovery^2^, was used in only 1.9% of cases. This is probably due to the high implantation cost, though its average hospitalization cost was similar to the other procedures. It would be interesting to increase investment with the implementation of this technology, despite the greater expense in the short term, as it reduces the average hospitalization time and indirect costs, allowing the young and economically active population undergoing this procedure to return to work faster, reducing hospital, business, and social security costs^24^.

Our study presents results similar to those observed by Carvalho et al.^25^, in an article published in 2019. The prevalence of obesity has shown an increasing trend throughout the country and in several segments of the Brazilian population^5^ ^,^ ^26^, and as bariatric surgery is a very important tool for the treatment of morbid obesity, the trend of its performance must be growing in a system that proposes to be structured according to the needs of its users. However, this was not true for most FUs, as most states showed a stationary trend, with three states showing a decreasing trend. Because they are complex procedures, with risks of complications (including death), which require specialized labor, hospitalizations, and supplies, many places in the country are unable to offer this service adequately. Thus, many people face difficulties in accessing health care and, although they are indicated for treatment, they cannot undergo it. This difficulty is related to the problems of the SUS’s poor budget management, which have been worsening after the Constitutional Amendment 95, of December 15, 2016, preventing a real increase in investment in health for 20 years^27^.

Paraná was the most prominent FU, with almost half of all surgeries, as well as showing a growing trend. This behavior can be explained by the great efficiency in performing bariatric surgery in the state, which allows greater access for the population to this procedure. The state is the second with the highest number of digestive system surgeons in the entire country, and the South is the region with the highest increase in this figure between 2011 and 2018^28^ ^,^ ^29^.

Finally, although this study is important to elucidate the spatial and temporal distribution of bariatric surgeries in Brazil, in addition to describing the trends of these procedures, it has limitations. By using secondary data, our work is susceptible to collection and reporting biases, which we could not mitigate. In addition, the study is limited to the information available, and the authors have no access to further details of the procedures performed. As it is an ecological study, it is also not possible to assess cause and effect relationships, as the cases are not analyzed individually, making it impossible to control for confounding factors. Finally, the deaths occurred during the periods of hospitalization, and it is not possible to assert that they were directly related to the specific procedure performed.

We described the characteristics and time series analysis of bariatric surgeries performed by SUS nationwide. We observed a predominance of females and white people undergoing the procedure. The trends are mostly stationary, but the country trend is increasing. We found an evident disparity between regions, suggesting access deficiencies in the country.

Despite the growth of obesity in Brazil and the expressive numbers related to morbid obesity, access to bariatric surgery is still very precarious, requiring better public policies and handling of socioeconomic and cultural factors. These actions are important to meet the demand and implement the principles and guidelines of the Unified Health System. By demonstrating which FUs and which demographic characteristics have lower rates of bariatric surgery, our study can direct public policies towards a more egalitarian Brazilian public health.
